# Establishment and validation of a prediction model for the first recurrence of Budd–Chiari syndrome after endovascular treatment: a large sample size, single-center retrospective study

**DOI:** 10.1007/s12072-022-10464-y

**Published:** 2022-12-26

**Authors:** Zhongkai Wang, Ziwei Wang, Zhiyuan Zhang, Jiandong Li, Zhiyang Pan, Ang Liu, Jian Lu, Jinhe Guo, Maoheng Zu, Hao Xu

**Affiliations:** 1 Department of Interventional Radiology, The Affiliated Hospital of Xuzhou Medical University, 99 West Huaihai Road, Xuzhou, 221006 Jiangsu China; 2 Department of Urology, Changhai Hospital, Naval Medical University, 168 Changhai Road, Shanghai, 200433 China; 3 Department of Interventional Oncology, The Second Affiliated Hospital of Xuzhou Medical University, 32 Meijian Road, Xuzhou, 221006 Jiangsu China; 4 Center of Interventional Radiology and Vascular Surgery, Department of Radiology, Zhongda Hospital, Medical School, Southeast University, Nanjing, 210009 Jiangsu China; 5 Department of Structural Heart Disease, Cardiovascular Institute and Fuwai Hospital, National Center for Cardiovascular Diseases, Chinese Academy of Medical Sciences and Peking Union Medical College, 167 Beilishi Road, Beijing, 100037 China

**Keywords:** Budd–Chiari syndrome, Endovascular treatment, Recurrence, Prediction model

## Abstract

**Objective:**

To investigate the independent risk factors for the first recurrence after endovascular management in patients with Budd–Chiari syndrome (BCS), and to establish a prediction model for predicting recurrence in target patients.

**Methods:**

BCS patients who underwent endovascular treatment in the Affiliated Hospital of Xuzhou Medical University from January 2010 to December 2015 were retrospectively examined, with their clinical, laboratory test, and imaging data collected and analyzed. Independent risk factors for recurrence were identified, and a prediction model was established and validated.

**Results:**

A total of 450 patients met the filtering criteria, and 102 recurred during the follow-up. The median follow-up time was 87 months, ranging from 1 to 137 months. The 1-, 3-, 5- and 10-year cumulative recurrence rate was 9.11% (6.41–11.73%), 17.35% (13.77–20.78%), 20.10% (16.30–23.72%), and 23.06% (18.86–27.04%), respectively. Liver cirrhosis, ascites, thrombosis, and all the main intrahepatic drainage veins obstructed (obstructed HV + AHV) are independent risk factors, while age is an independent protective factor. The prediction model was named MRBET. Based on the model, the risk score of each patient equals (−0.385981 * Age/10) + (0.0404184 * PT) + (0.0943423 * CRE/10) + (0.0157053 * LDH/10) + (0.592179 * LC) + (0.896034 * Ascites) + (0.691346 * Thrombosis) + (0.886741 * obstructed HV + AHV), and those in the high-risk group (risk score ≥ 1.57) were more likely to recur than those in the low-risk group (HR = 6.911, *p* < 0.001). The MRBET model is also available as a web tool at https://mrbet.shinyapps.io/dynnomapp.

**Conclusion:**

Liver cirrhosis, ascites, thrombosis, and obstructed HV + AHV are independent risk factors for the first recurrence; age is an independent protective factor. The prediction model can effectively and conveniently predict the risk of recurrence and screen out patients at a high recurrence risk.

**Supplementary Information:**

The online version contains supplementary material available at 10.1007/s12072-022-10464-y.

## Introduction

Budd–Chiari syndrome (BCS) is characterized by obstruction at any level from the hepatic veins (HV) to the inferior vena cava (IVC) outflow [1]. In Western countries, BCS is a rare disorder that principally results from thrombosis, whose etiology has been ascribed to several factors including myeloproliferative neoplasms (MPNs), antiphospholipid syndrome, paroxysmal nocturnal hemoglobinuria (PNH), antithrombin deficiency, etc. [2–4]. In contrast, although there are over twenty thousand reported cases in China, the aforementioned risk factors are not common [5, 6]. Therefore, in the West, anticoagulation or TIPS is effective, while angioplasty merely works in a minority of cases [3–7]. In the Asia–Pacific region, symptomatic BCS with membranous or segmental obstruction accounts for a relatively high proportion, and angioplasty could benefit patients to the greatest extent regardless of stent placement [8, 9].

Over the decades, with the progress and maturity of endovascular treatment against BCS, the prognosis is generally favorable except for a fraction of patients with fulminant, acute liver failure, or other significant complications [10, 11]. Considering the favorable prognosis and chronic processing of the disease in most Chinses patients, concerns have recently been focused on those who suffer from repeated recurrence after treatment [11]. Let alone poor prognosis itself is associated with untreated recurrence [12].

Nonetheless, to the best of our knowledge, few studies have been conducted on risk factors for the recurrence of BCS. Because of the rarity of the disease, cohort studies with large sample sizes are even few and far between. This study aims to identify the independent risk factors for the first recurrence of BCS after endovascular treatment, as well as to establish and validate a prediction model and nomogram which could distinguish the risk of recurrence in patients through the analysis of 450 cases.

## Patients and methods

### Patients

In our study, patients with BCS who prepared for endovascular treatment were consecutively admitted to our hospital from January 2010 to December 2015. Their clinical, laboratory test, and imaging data were collected and retrospectively analyzed. The exclusion criteria were: 1. patients who have previously been diagnosed and received medical, surgical, endovascular treatment, or TIPS; 2. hepatic outflow obstruction caused by congestive heart disease, sinusoidal obstruction syndrome, or other causes; 3. significant dysfunction of vital organs such as liver, kidney, and brain; 4. secondary BCS; 5. recanalization procedure failed due to complete occlusion or complicated with old thrombi of vessel lesions; 6. patients with irregular and unstandardized anticoagulation.

Our principle of endovascular treatment is to recanalize as many veins as possible. For patients with a concurrent IVC obstruction, IVC recanalization is usually first performed. When HV recanalization was technically challenging, high risk, and could fail, the selective recanalization of the obstructed large accessory hepatic vein (AHV) (if any) could achieve an expected intrahepatic drainage effect. We applied a stepwise strategy during the procedure, with initial balloon dilation, followed by stenting when the obstructed lumen retracted > 75% or the cross-lesion pressure difference was ≥ 4 cmH_2_O after repeated dilation.

The primary endpoint of the study was the first recurrence after endovascular treatment. Recurrence was defined as a stenosis or occlusion in HVs, IVC or collateral veins after endovascular treatment, or relevant clinical symptoms that appear after a steady condition. All patients were followed up every 3 to 6 months from the date of diagnosis until study closure (December 31, 2020), or the death of patients, the date of the last follow-up. The state of and the duration before the first recurrence were determined by telephone follow-up and/or outpatient records. Enrolled patients were assigned to two groups: the recurrence group and the non-recurrence group.

### Clinical assessment

Variables used in the analysis were selected based on the representative parameters in BCS, and relevant factors for recurrence reported previously, including gender, age, laboratory data, clinical characteristics, vascular involvement, Child–Pugh score, model of end-stage liver disease (MELD) score, and BCS-specific prognostic indices.

The criteria of diagnosis followed the BCS diagnosis and treatment specifications [1, 2]. Diagnosis was made in our center through color Doppler ultrasonography (CDUS), computed tomography (CT), magnetic resonance imaging (MRI), and/or venography. Therefore, the first available data after a definite diagnosis were used as the baseline data. Clinical characteristics, including hepatocellular carcinoma (HCC), upper gastrointestinal bleeding (UGB), liver cirrhosis (LC), and ascites, were examined by radiology or endoscopy, while hepatic encephalopathy (HE) was evaluated by the West Haven scale. The vascular involvement was evaluated by (1) whether the main intrahepatic drainage veins are obstructed, (2) whether the IVC is obstructed, and (3) whether the involved veins are complicated with thrombosis. The main intrahepatic drainage veins obstruction was further subclassified as (1) all the main intrahepatic drainage veins were obstructed; (2) at least one main intrahepatic drainage vein was patent. The main intrahepatic drainage veins include three main hepatic veins (left, middle, and right HV) and large patent AHVs (if any). AHV is defined as a HV with a diameter ≥ 5 mm in the third portal hilum [13, 14]. Child–Pugh score, MELD score, and BCS-specific scores (Clichy PI and Rotterdam BCS index) were calculated as reported [7, 15–17].

### Statistical analysis

The modeling process is summarized in Supplementary Fig. 1. The primary endpoint of interest is the first recurrence time after endovascular treatment. Multiple strategies were applied to ensure reliable estimation of the variable effect in fitting the global model, and variables (1) presenting strong collinearity (|*r*|≥ 0.5); (2) with low occurrence (HE & HCC); (3) derived from individual variables (Child–Pugh score, MELD score, Clichy PI, and Rotterdam BCS index); (4) with a *p* value > 0.2 in the univariate screening were excluded.

The global multivariate Cox regression model was fitted with all variables that passed filtering. Age, ALT, total bilirubin (TBIL), creatinine (CRE), albumin (ALB), lactate dehydrogenase (LDH), and gamma-glutamyl transpeptidase (GGT), were scaled down by a factor of 10 for better interpretation of the estimated effect. The reduced model was constructed through backward eliminations (BE) of the global model, with the Akaike information criterion (AIC) as the stopping rule. The modeling stability was evaluated with 1000 times bootstrap. The C-statistics and calibration curve were, respectively, applied to measure the discriminative and calibrating competence of the model. C-statistics was adjusted with rms::validate() to alleviate optimism. The risk score of each patient is calculated by a formula constructed from the variables in the reduced model. The coefficient of each variables was extracted using the stats::coef() function. The risk score calculator based on the formula is provided in Supplementary Table 1 for easy application. The survminer::surv_cutpoint() function was used to determine the optimal cutoff of the risk score for risk stratification. The model was presented as both a regression formula and a nomogram. The web tool was built and deployed to shinyapp.io using the DynNom package. All statistical analyses were performed with R software (version 4.0.3), and the significant level (α) was set at 0.05 for all statistical tests for significance.

## Results

### Patient characteristics and follow-up results

**Fig. 1:**
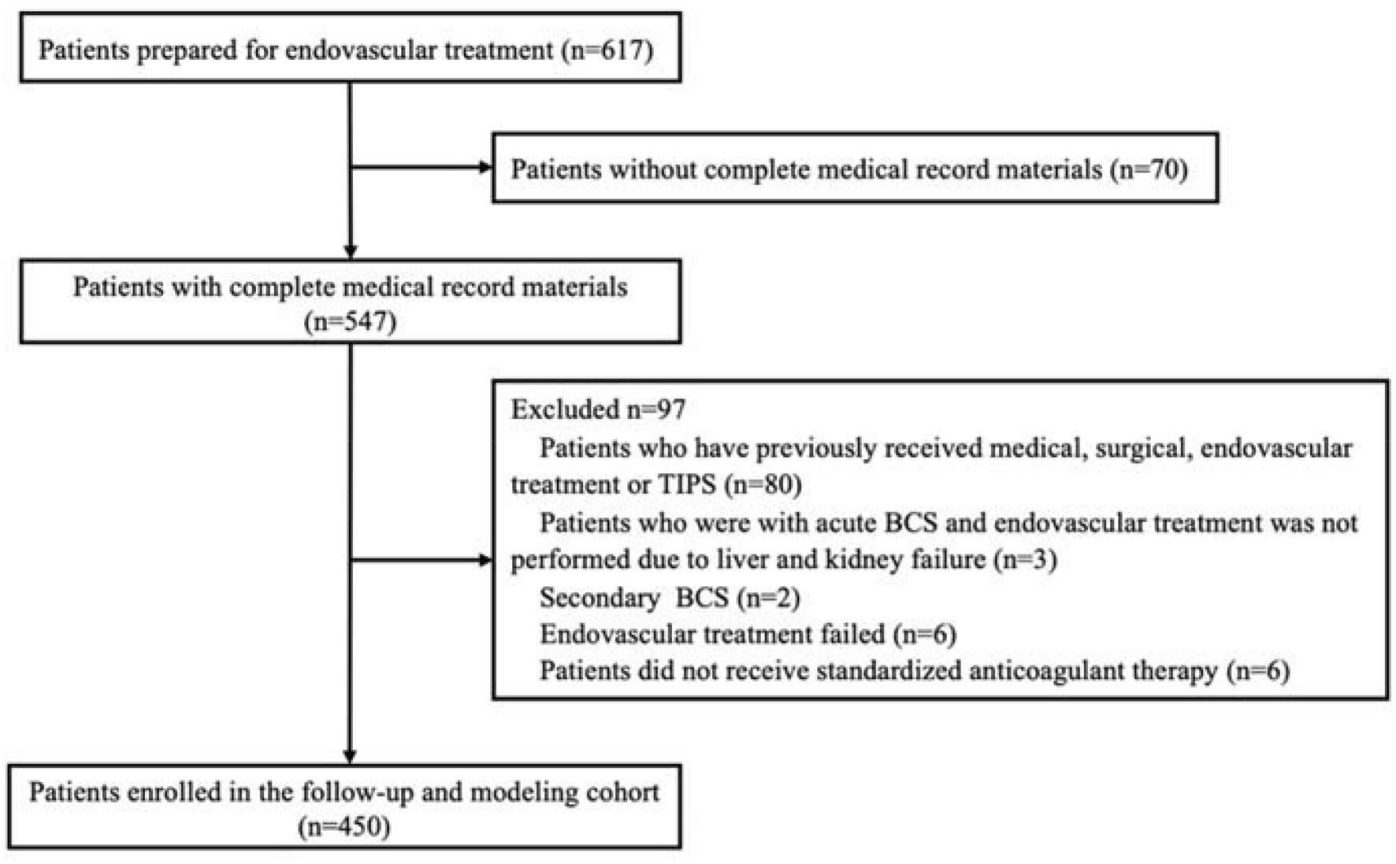
Flowchart of this study

From January 2010 to December 2015, 617 BCS patients were admitted to our center and planned for endovascular treatment. Complete medical record materials of 547 patients could be retrieved. Of these patients, 80 had previously undergone surgery, endovascular treatment, or TIPS, 3 had canceled endovascular treatment due to liver and kidney failure caused by acute BCS, and 2 had secondary BCS caused by liver metastatic tumor-induced HV compression. Also, endovascular treatment failed in six patients, including three cases with whole-range occlusion of IVC, two cases complicated with old IVC thrombus, and one case with hepatic vein atrophy. Moreover, six cases did not receive standardized anticoagulant therapy according to medical advice. Finally, a total of 97 patients were excluded resulting in 450 patients included for modeling. The flowchart of this study is shown in Fig. 1.

During the follow-up period, 21 patients were lost to follow-up, and 32 patients died before recurrence. Of the dead patients, 19 were complicated with HCC at admission or newly developed HCC after discharge, 3 died of UGB, 3 died of severe hepatic encephalopathy, 1 died of lung cancer, 1 died of esophageal cancer, and 1 died of cerebral infarction.

**Fig. 2:**
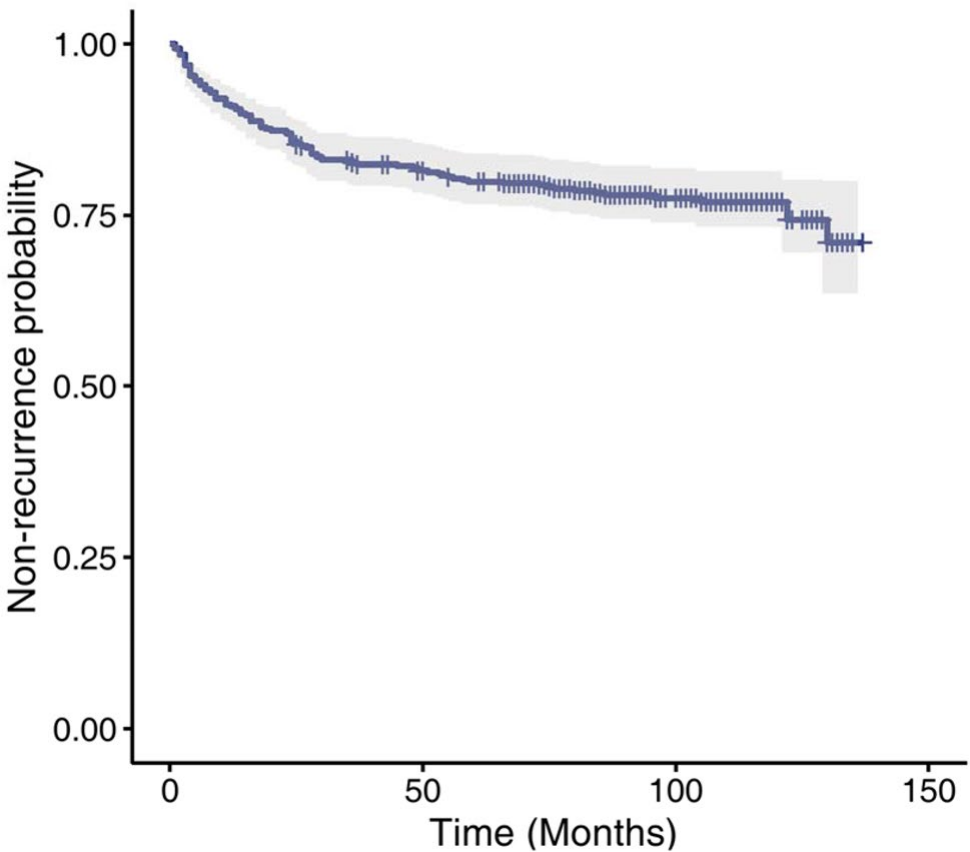
Kaplan–Meier survival curve of the study cohort (*n* = 450) with recurrence as the end point of follow-up

The median follow-up time was 87 months, ranging from 1 to 137 months. The 1-, 3-, 5-, and 10-year recurrence rate was 9.11% (6.41–11.73%), 17.35% (13.77–20.78%), 20.10% (16.30–23.72%), and 23.06% (18.86–27.04%), respectively (Fig. 2). Notably, those patients who had a recurrence within 5 years after treatment accounted for 74.51% (76/102) of all the recurrent patients. Only 7.0% of patients who were followed up for more than 5 years had a recurrence. The difference between 3-, 5-, and 10-year recurrence rates showed no statistical significance (all *p* > 0.05). The baseline characteristics of the recurrence and the non-recurrence group are summarized in Table 1.

**Table 1:** Baseline characteristics in the study cohort

Characteristics	Non-recurrence (*n* = 348)	Recurrence (*n* = 102)	*p* value
Female (*n*, %)	174 (50%)	60 (58.82%)	0.145
Age (years)	48 (41–57)	41 (30.2–49)	< 0.001
AST (U/L)	26.5 (21–33)	31.5 (24–42)	< 0.001
ALT (U/L)	19 (14–25.2)	25 (18–33)	< 0.001
Total bilirubin (μmol/L)	27 (16.7–37.5)	29.6 (22.4–51.2)	0.002
Creatinine (μmol/L)	58 (49–67)	60 (53–70.8)	0.036
Albumin (μmol/L)	40.8 (36.7–44.2)	38 (32–43.5)	< 0.001
Sodium (mmol/L)	140.9 (139.2,142.7)	140.1 (137.9–142.2)	0.012
LDH (U/L)	179.5 (154.8–211.2)	189 (166.2–236.5)	0.005
ALP (U/L)	94 (71–119.2)	116.5 (82.2–150.5)	< 0.001
GGT (U/L)	74 (42–122.2)	94 (60.2–138)	0.009
Platelet (10^9^/L)	96 (70.8–135.2)	108 (76–165)	0.086
PT (seconds)	14.5 (13.3–15.8)	15.2 (14.4–17.4)	< 0.001
INR	1.2 (1.1–1.3)	1.3 (1.2–1.4)	< 0.001
HCC (*n*, %)	6 (1.72%)	1 (0.98%)	0.937
UGB (*n*, %)	18 (5.17%)	18 (17.65%)	< 0.001
Liver cirrhosis (*n*, %)	76 (21.84%)	37 (36.27%)	0.005
Ascites (*n*, %)	150 (43.1%)	81 (79.41%)	< 0.001
HE (*n*, %)	3 (0.86%)	0 (0%)	0.803
Thrombosis (*n*, %)	54 (15.52%)	33 (32.35%)	< 0.001
Obstructed IVC (*n*, %)	298 (85.63%)	73 (71.57%)	0.002
Obstructed HV + AHV (*n*, %)	16 (4.60%)	24 (23.53%)	< 0.001
Child–Pugh score	6 (5–7)	8 (6–9)	< 0.001
Child–Pugh grade (*n*, %)			< 0.001
A	215 (61.78%)	31 (30.39%)	
B	116 (33.33%)	51 (50%)	
C	17 (4.89%)	20 (19.61%)	
MELD score	4.5 (2.4–8.2)	8.3 (4.5–11)	< 0.001
Clichy PI score	5.3 (4.6–6)	5.5 (4.8–6)	0.211
Rotterdam score	0.2 (0.1–1.1)	1.1 (1.1–1.2)	< 0.001
Rotterdam grade (*n*, %)			< 0.001
I	218 (62.64%)	25 (24.51%)	
II	123 (35.34%)	74 (72.55%)	
III	7 (2.01%)	3 (2.94%)	

*AST* aspartate aminotransferase, *ALT* alanine aminotransferase, *LDH* lactate dehydrogenase, *ALP* alkaline phosphatase, *GGT* gamma-glutamyl transpeptidase, *PT* prothrombin time, *INR* international normalized ratio, *HCC* hepatocelluar carcinoma, *UGB* upper gastrointestinal bleeding, *HE* hepatic encephalopathy, *IVC* inferior vena cava, *HV* hepatic vein, *AHV* accessory hepatic vein

### Prediction model

**Fig. 3:**
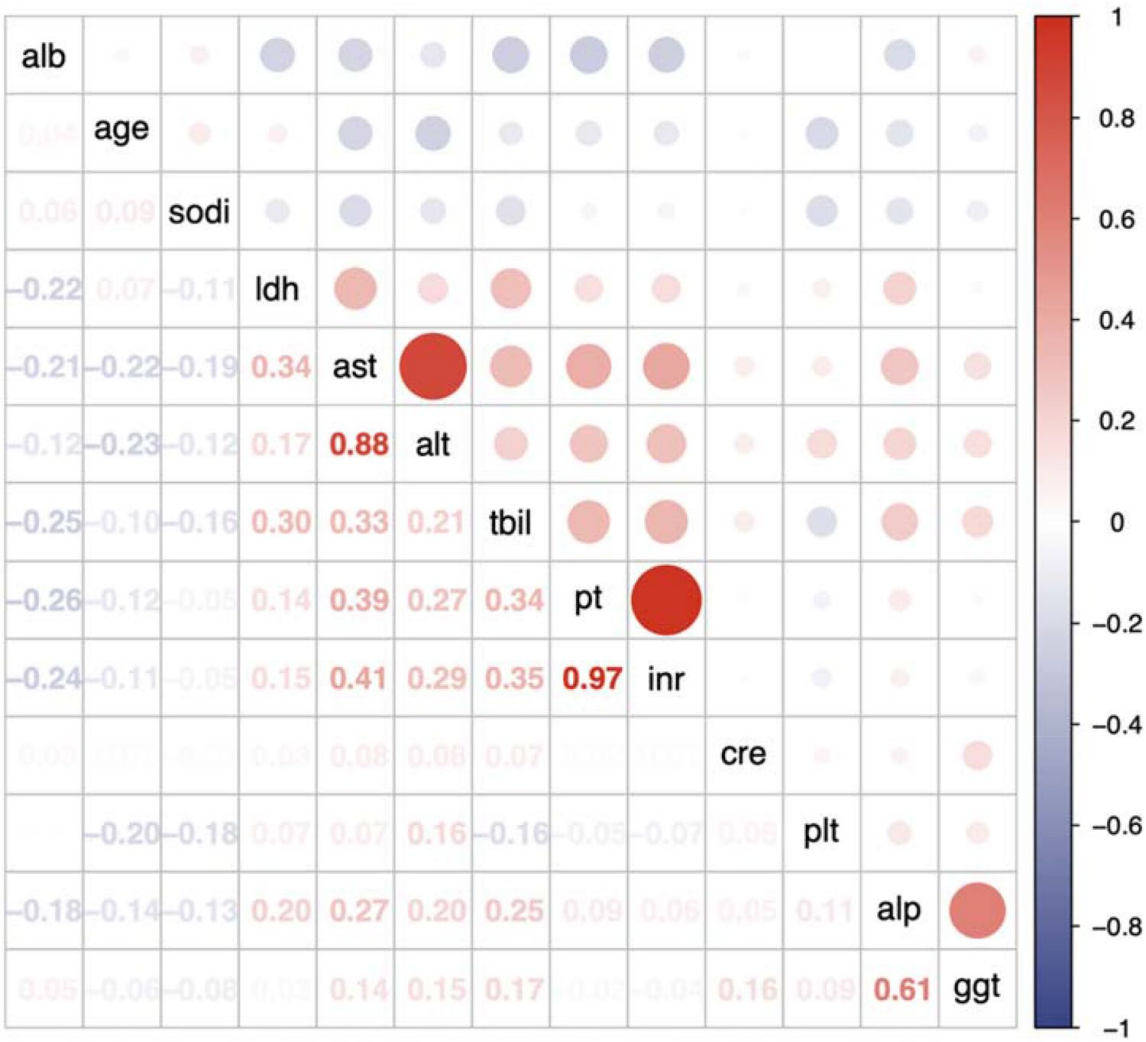
Result of the analysis of collinearity between continuous variables preparing to be enrolled in the model

**Fig. 4:**
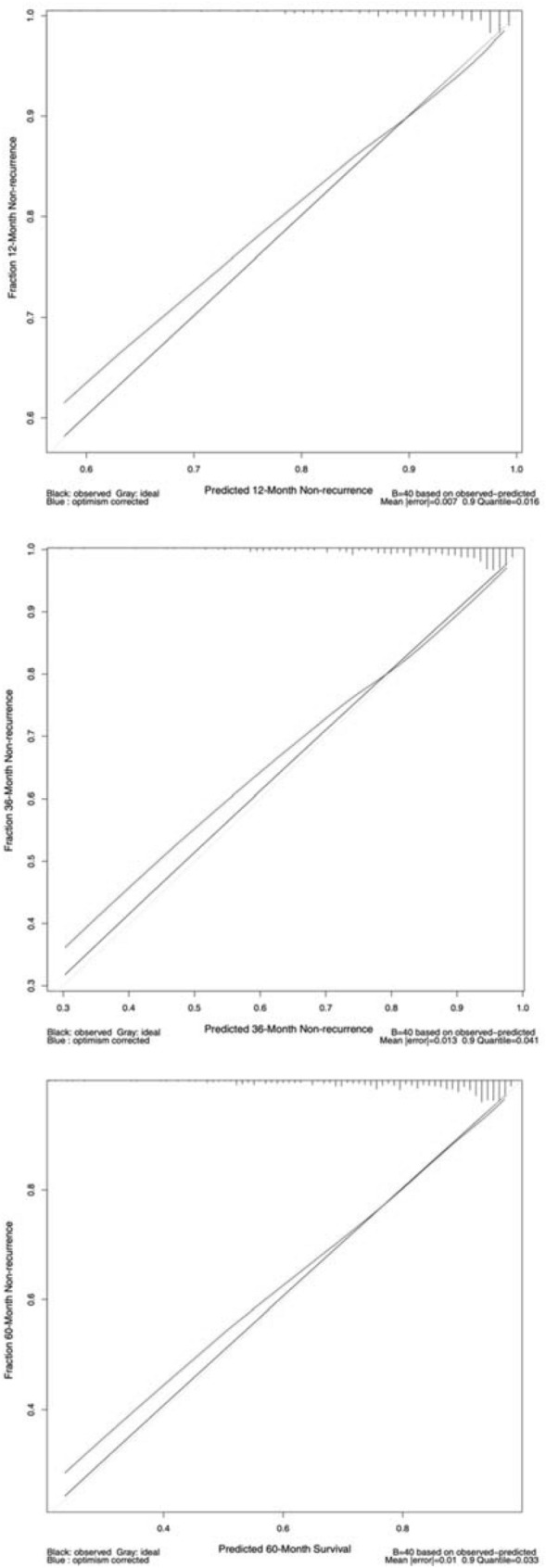
Calibration curve at 1-, 3-, and 5-year of the recurrence model

After the univariate screening (see “Patients and methods”, Fig. 3, Table 2), the global model was fitted with age, PT, ALT, PLT, TBIL, CRE, ALB, LDH, GGT, gender, LC, UGB, ascites, thrombosis, IVC, and all the main intrahepatic drainage veins obstructed (obstructed HV + AHV). Stepwise backward elimination chose the optimal model with reduced variables. The model development progress is summarized in Supplementary Fig. 1 and Table 2. In the reduced model, LC, ascites, thrombosis, and obstructed HV + AHV are independent risk factors while age is an independent protective factor. The effect of CRE (*p* = 0.105), PT (*p* = 0.099), and LDH (*p* = 0.119) is not significant. After internal validation using 1000-time bootstrap, the optimism-corrected C-index is 0.772, suggesting that the model has a good discriminating ability. Also, the calibration curve at 1-, 3-, and 5-year showed good calibration (Fig. 4). To quantify the patients’ risk of recurrence, we generated a formula to calculate the risk score of each patient (see “Patients and methods”): risk score = (−0.385981 * Age/10) + (0.0404184 * PT) + (0.0943423 * CRE/10) + (0.0157053 * LDH/10) + (0.592179 * LC) + (0.896034 * Ascites) + (0.691346 * Thrombosis) + (0.886741 * obstructed HV + AHV), higher value suggests higher recurrence risk. LC, ascites, thrombosis of involved veins, and obstructed HV + AHV were all binary variables, with the value of 1 (present) and 0 (absent), respectively. The risk score could be calculated automatically using the Supplementary Table 1.

**Table 2:** Univariate and multivariate analysis for recurrence

Predictors	Univariate models			Multivariate model (global)			Multivariate model (reduced)		
	HR	95% CI	*p* value	HR	95% CI	*p* value	HR	95% CI	*p* value
Female	1.373	(0.926–2.037)	0.115	1.030	(0.651–1.629)	0.901			
Age (10 years)	0.602	(0.516–0.702)	< 0.001	0.695	(0.588–0.820)	< 0.001	0.680	(0.585–0.790)	< 0.001
PT (1 s)	1.096	(1.058–1.135)	< 0.001	1.035	(0.982–1.091)	0.197	1.041	(0.993–1.092)	0.098
Platelet (10^10^/L)	1.025	(1.000–1.051)	0.050	1.014	(0.984–1.045)	0.378			
ALT (10 U/L)	1.108	(1.070–1.147)	< 0.001	1.015	(0.967–1.065)	0.549			
Total bilirubin (10 μmol/L)	1.098	(1.029–1.173)	0.005	0.995	(0.912–1.087)	0.916			
Creatinine (10 μmol/L)	1.106	(0.992–1.233)	0.071	1.084	(0.955–1.229)	0.211	1.099	(0.980–1.232)	0.105
Albumin (10 g/L)	0.559	(0.419–0.746)	< 0.001	0.921	(0.647–1.312)	0.649			
LDH (10 U/L)	1.022	(1.006–1.039)	0.009	1.013	(0.989–1.036)	0.291	1.016	(0.996–1.036)	0.119
GGT (10 U/L)	1.014	(0.996–1.032)	0.117	1.011	(0.991–1.031)	0.284			
Liver cirrhosis	1.841	(1.228–2.759)	0.003	1.629	(1.005–2.640)	0.048	1.808	(1.184–2.761)	0.006
UGB	2.870	(1.723–4.780)	< 0.001	1.454	(0.800–2.642)	0.219			
Ascites	4.343	(2.686–7.020)	< 0.001	2.418	(1.404–4.163)	0.001	2.450	(1.470–4.084)	< 0.001
Obstructed IVC	0.470	(0.306–0.723)	< 0.001	1.121	(0.668–1.882)	0.665			
Obstructed HV + AHV	4.293	(2.715–6.791)	< 0.001	2.302	(1.335–3.969)	0.003	2.427	(1.435–4.105)	< 0.001
Thrombosis	2.289	(1.511–3.467)	< 0.001	1.873	(1.171–2.997)	0.009	1.996	(1.294–3.081)	< 0.001

*PT* prothrombin time, *ALT* alanine aminotransferase, *LDH* lactate dehydrogenase, *GGT* gamma-glutamyl transpeptidase, *UGB* upper gastrointestinal bleeding, *IVC* inferior vena cava, *HV* hepatic vein, *AHV* accessory hepatic vein

**Fig. 5:**
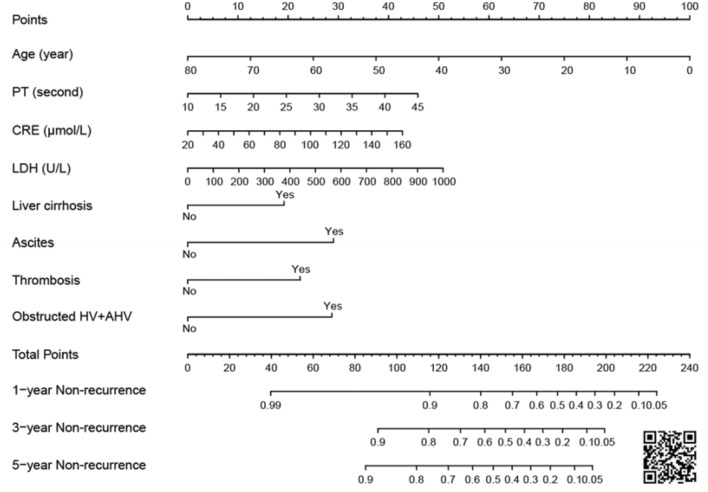
Nomogram for BCS recurrence after endovascular treatment. The online version is accessible by scanning the QR code

For better clinical application, a nomogram was provided to estimate non-recurrence probability (Fig. 5). In the nomogram, the corresponding score can be found for each variable, and the total score of patients can be summed up. Non-recurrence probability at different time points after endovascular treatment can be speculated with the corresponding probability of the total score. We named our model as MRBET, which is short for ‘Model for Recurrence of BCS after Endovascular Treatment’. The MRBET model was also presented as an easy-to-use web tool that is freely available at https://mrbet.shinyapps.io/dynnomapp. By providing all required predictors, the recurrence probability of future patients could be predicted at any given time point.

**Fig. 6:**
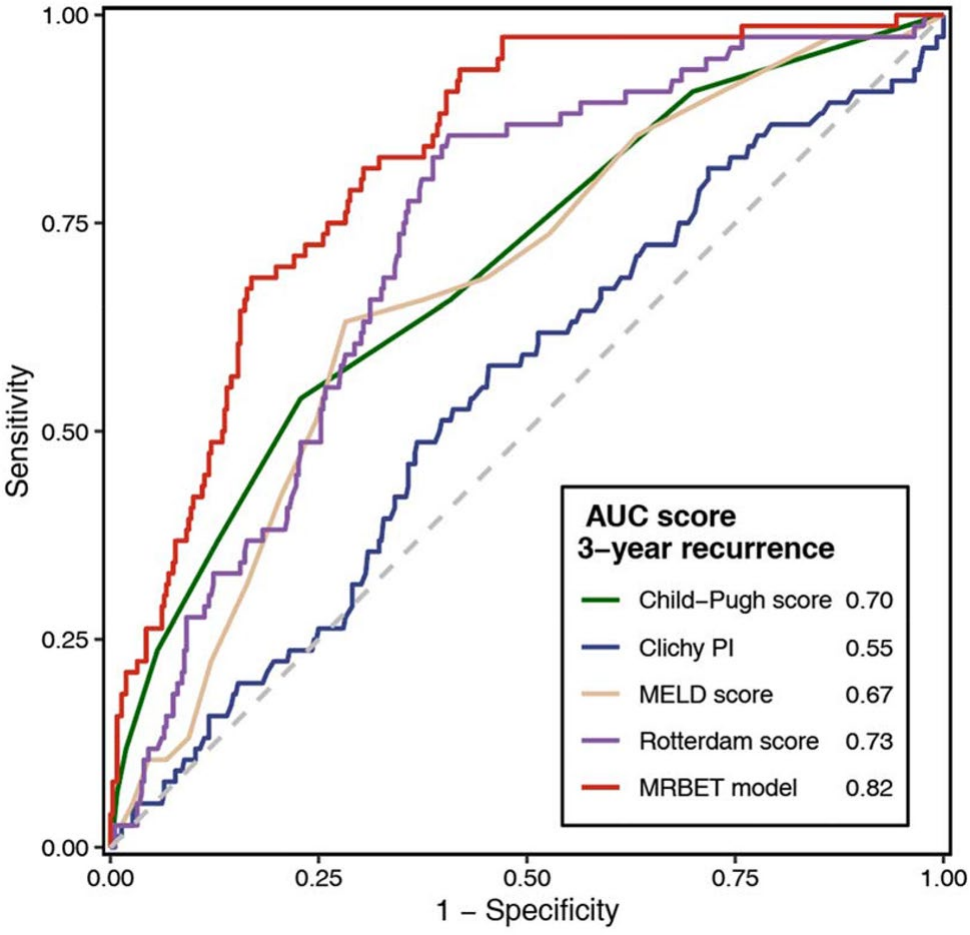
Time-ROC curves of the MRBET model and previous models in this study

Since this is the first study that focused on developing a prognostic model to predict the first recurrence of BCS patients after endovascular treatment, we compared this model with Child–Pugh score, MELD score, Clichy PI, and Rotterdam BCS index to justify the necessity of establishing a dedicated model. Time-ROC curves proved that the recurrence model developed in this study outperformed other non-dedicated models in predicting 3-year recurrence (Fig. 6). The area under curve (AUC) for predicting 3-year recurrence was 0.82, which was better than Child–Pugh score (0.70), Clichy PI (0.55), MELD score (0.67), and Rotterdam BCS index (0.73).

### Recurrence risk stratification

**Fig. 7:**
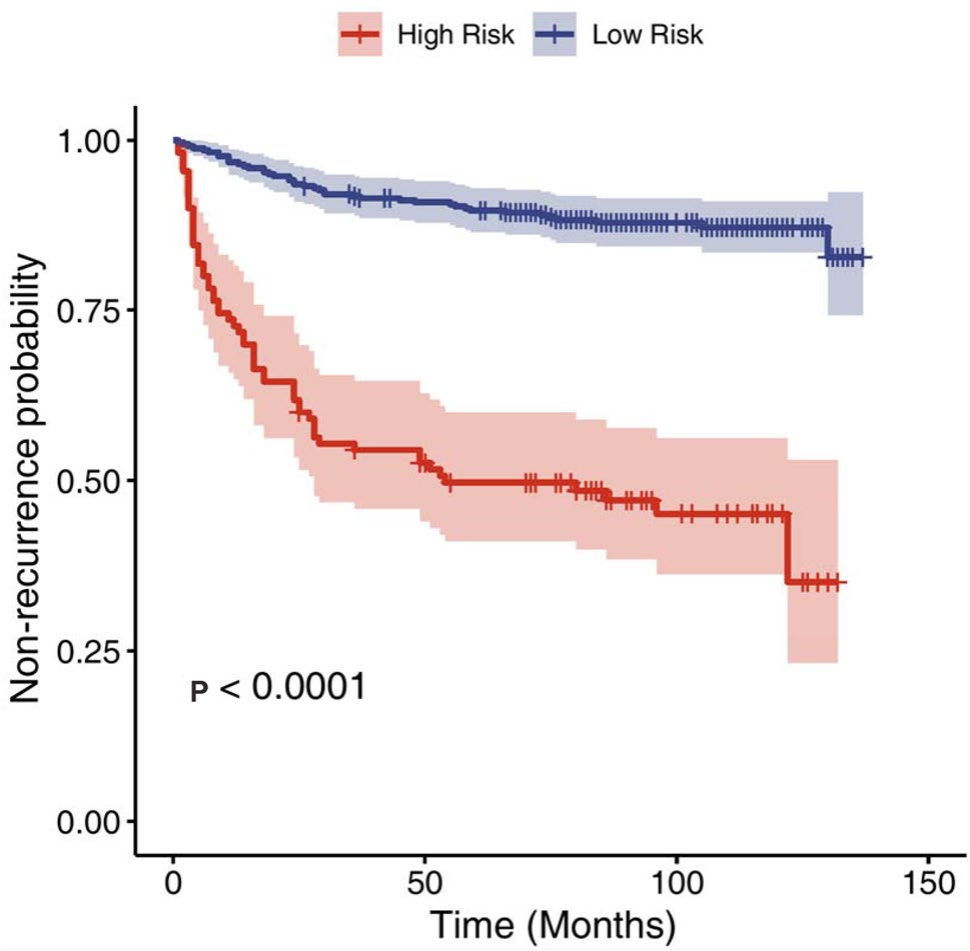
Recurrence risk stratification based on the risk score value

The risk score was calculated for each patient who accepted endovascular treatment based on the previously obtained formula and ranged from −1.25 to 4.41. The patients with risk score value < 1.57 were stratified as the low-risk group and ≥ 1.57 as the high-risk group (“Patients and methods”). The difference in recurrence risk between the two groups was statistically significant (HR = 6.911, *p* < 0.001) (Fig. 7). The 1-, 3-, and 5-year recurrence rate in low-risk group was 2.65% (0.93–4.35%), 7.97% (5.04–10.81%), and 10.08% (6.81–13.24%), compared with 28.83% (19.88–6.78%), 46.03% (35.9–54.56%), and 50.87% (40.54–59.41%) in high-risk group.

## Discussion

China has the highest number of diagnosed BCS patients globally, with at least 1,900 pieces of literature reporting more than 20,000 cases. However, prevalent risk factors reported in the West are relatively rare in Chinese patients [5, 6, 18]. Hence, discrepancies in clinical manifestations and treatment options of BCS exist between the two regions. Despite more than half of patients in the West being complicated with HV thrombosis, membranous or segmental obstruction is the most common in the Asia–Pacific region, which provides an opportunity to restore intrahepatic venous drainage through endovascular recanalization [8, 11, 12, 19].

In recent years, the development of endovascular treatment and materials, supported by extensive evidence-based medicine, has furthered the understanding of BCS among the physician community and improved the outcome of BCS. A meta-analysis of 2,255 patients by Zhang et al. suggested that the 1- and 6-year survival rates of patients receiving endovascular treatment were 92% (89.8–94.3%) and 76.4% (72.4–80.5%), were 87.3% (83.2–91.3%) and 72.1% (67.2–77.0%) after TIPS, respectively [20]. Meanwhile, a variety of models have been established to predict patients’ prognoses [7, 17, 21–23]. Unfortunately, although managing recurrent patients has constituted most of the clinical workload, few studies have been conducted on BCS recurrence, especially ones with large sample size. Additionally, Han et al. confirmed that untreated recurrence was closely associated with poor prognosis [12].

In a study involving 143 BCS patients, Cui et al. found that the 1-, 3-, and 6-year initial patency rate after endovascular treatment was 91.1%, 77.4%, and 74.0%, respectively [24]. Another study involving 177 patients showed cumulative 1-, 5-, and 10-year initial patency rates of 95%, 77%, and 58%, respectively [12]. The 1-, 3-, 5- and 10-year cumulative first recurrence rate in our study was 9.11% (6.41–11.73%), 17.35% (13.77–20.78%), 20.10% (16.30–23.72%), and 23.06% (18.86––27.04%), respectively, consistent with previous studies. It is worth mentioning that the difference between the 3-year and the 5- or 10-year recurrence rate was not statistically significant (all *p* < 0.05). Therefore, we suggest that the first recurrence peak after treatment is mainly within the first 3 years. Patients with no recurrence for more than 3 years are less likely to have disease progression. Compared with previous studies, the 5- and 10-year recurrence rates in this study were lower. We cautiously consider the first recurrence peak period in the first 3 years may also be that, despite the large sample size of our study, the number of cases with long-term recurrence was still limited, resulting in a wide confidence interval (95%CI) and no statistically significant difference was observed.

In the final multifactor model, liver cirrhosis, ascites, thrombosis, and obstructed HV + AHV are independent risk factors, while age is an independent protective factor (all *p* < 0.001). All factors included in the model could be easily obtained at the time of diagnosis, considering the feedback from the actual clinical application of some previous specific prognostic models. For instance, both Clichy PI and New Clichy PI include the clinical effect of ascites to treatment, thus impeding its use at the first diagnosis.

Patients under 30 were at higher risk of recurrence according to a study involving 471 cases between 2008 and 2012 [25]. Wang et al. demonstrated that patients aged 5 to 29 with HV involvement had the highest recurrence rate [26]. A large-scale retrospective cohort study by Li et al. also confirmed that age was a significant risk factor for recurrence after endovascular treatment in patients with IVC involvement [27]. Meanwhile, in Clichy/New Clichy PI, Rotterdam BCS index, and BCS-TIPS score, age is also included as a component [7, 17, 21, 22]. The observation above was also confirmed in our study. Nonetheless, the underlying mechanism of how age plays a protective role as an independent factor needs to be discovered.

We concluded that liver cirrhosis is an independent risk factor, consistent with a single-center study involving 130 BCS patients in China [28]. We speculate that the influence of liver cirrhosis on patients’ recurrence may be related to the following reasons: (1) hemodynamic changes: cirrhosis is characterized by diffuse proliferation of fibrous tissue. Relative stasis of blood flow in portal and hepatic venous system leads to thrombosis [29]. (2) Vascular endothelial damage: hemorrhagic cirrhosis caused by BCS results in severe congestion of internal organs, increased shear stress in the vascular wall, and disruption of the mucosal barrier of the digestive tract. Consequently, bacteria and toxins entering the circulation damage the vascular endothelium, which exposes subcutaneous tissue and activates the coagulation pathway, accelerating thrombosis in vessels or stents [30]. (3). Blood hypercoagulable state: recent studies have shown that the rebalancing blood coagulation system in patients with cirrhosis is quite fragile and can tilt toward either state of bleeding or thrombosis. The increased production of vWF and fibrinogen, changes in fibrin structure, and a low fibrinolysis state all lead to a high risk of thrombosis. This phenomenon has no significant statistical difference between liver cirrhosis with different etiology [31, 32].

Ascites, a traditional and classic indicator, is universal in predicting disease outcomes in patients with liver disease, which has been confirmed by many studies [7, 17, 21]. In our study, it is also associated with the first recurrence of patients. The presence of ascites often implies worse liver function and more severe venous obstruction, as mentioned earlier, which contributes to the recurrence.

Thrombotic events represent the progression of patients from a thrombophilic state. Under this circumstance, multiple veins are usually involved, with more distinct clinical manifestations and serious hepatic injury, leading to BCS recurrence in 5–11% of cases [33, 34]. Extensive screening for thrombogenic factors is not recommended in China according to current guidelines. But for patients with thrombosis, detection of MPNs and its related genes such as JAK2V617F, coagulation factor V Leiden, thrombin G20210A, PNH, MTHFR gene, protein C and S, and other factors is reasonable.

Obstruction of all the main intrahepatic drainage veins is an independent risk factor for recurrence. In 1952, Elias and Petty reported the existence of lower HVs outside the second hepatic portal [35]. Afterward, HVs were divided into superior and inferior groups [36]. The superior group consists of three main branches: the left, middle, and right HV, which flowed into the IVC through the second hilum. The venous trunk of the inferior group refers to as the AHV, including the caudate lobe vein and inferior right HV, which merge into the IVC through the third hilum. Caudate lobe veins are often small and undetectable, while the inferior right HV is sometimes large, which is magnitude in liver surgery and interventional procedures [37]. When BCS occurs with main HVs partially or completely obstructed, hepatic hypertension arises. In this case, AHVs sometimes compensate for dilation and act as a bridge between the portal vein (PV) and IVC to fulfill the intrahepatic drainage [38]. Plentiful studies in the past decade have confirmed that the recanalization of obstructed AHV can effectively relieve hepatic congestion and reduce liver function injury and PV pressure [39, 40]. When the main intrahepatic drainage veins, including three main HVs and AHVs (if any), are obstructed, congestive liver injury and cirrhosis aggravate, increasing the recurrence risk of patients. Generally, under this circumstance, we would manage to treat all diseased veins we observed through angiography. When there are still one or two remaining stenotic veins with unsatisfied balloon angioplasty and difficult stent placement, the intrahepatic venous drainage could still be fulfilled because most veins have been recanalized. This situation is quite common when all the main intrahepatic drainage veins are obstructed. However, the stasis of blood flow in the untreated veins results in hemodynamic changes and, ultimately, an increased risk of thrombosis. We speculate that this is one of the causes of secondary venous thrombosis and occlusion, which differs from the primary thrombophilic state in patients due to genetic mutations. There may be potential interaction between various thrombosis causes, which needs further exploration. In brief, these findings reemphasize the importance of fully recanalizing as many veins as possible.

PT, CRE, and LDH were selected by AIC and tended to be risk factors for BCS recurrence in the multivariate modeling. Although not statistically significant in our analysis, PT and CRE have repeatedly appeared in prognostic indices for BCS [7, 17], and LDH was considered an independent risk factor for BCS recurrence in a previous retrospective study [25]. Prolongation of PT reflects poor liver function, and it suggests, in part, that the fragile rebalancing blood coagulation system in BCS patients could sometimes tilt towards a state of bleeding, as mentioned in the “Discussion” [31, 32]. Despite its limitations, baseline CRE is still the most used biomarker for estimating glomerular filtration rate and assessing kidney injury in patients with cirrhosis [41]. Increased CRE implies poor liver function and thus potentially affects patients’ recurrence. LDH is an essential enzyme in the glycolytic pathway, released due to body tissue damage. The liver injury could promote LDH activities. Moreover, experiments based on large samples demonstrated higher LDH levels in MPNs and PNH, while the etiology of BCS has been ascribed to several factors, including MPNs and PNH [42, 43]. Further studies with larger sample sizes are needed to validate their clinical value.

Our prediction model, as described in “Results”, has good discrimination and calibration in predicting the first recurrence of patients with BCS after endovascular treatment, and convenient application, promising future popularization.

This study still has some limitations: (1) as a retrospective study with a long time span, recall bias will inevitably occur; (2) Although our center attracts patients from all over the country, more than half of the patients are still confined to the provincial area; the single-center research led to an unavoidable geographical shift; (3) thrombogenic factors such as JAK2V617F and MTHFR mutation were not included mainly due to the insufficient detection in our follow-up samples; and (4) there is still a lack of external validation. At present, studies on BCS with large sample size in China are only carried out by a few centers independently; multi-center cooperation is imperative.

In conclusion, liver cirrhosis, ascites, thrombosis, and obstructed HV + AHV are independent risk factors for the first recurrence of BCS patients after endovascular treatment. The prediction model can effectively and conveniently predict the risk of recurrence and screen out patients at a high recurrence risk.

## Supplementary Information

Below is the link to the electronic supplementary material.

Supplementary file1 (DOCX 77 KB)

Supplementary file2 (XLSX 11 KB)

## Data Availability

Data available on request from the authors.
