# Supplementary figures and images for: Establishment and validation of a prediction model for the first recurrence of Budd–Chiari syndrome after endovascular treatment: a large sample size, single-center retrospective study
Source: Hepatol Int. 2022 Dec 26;17(1):159–69. doi: 10.1007/s12072-022-10464-y (PMC9895038; doi:10.1007/s12072-022-10464-y)

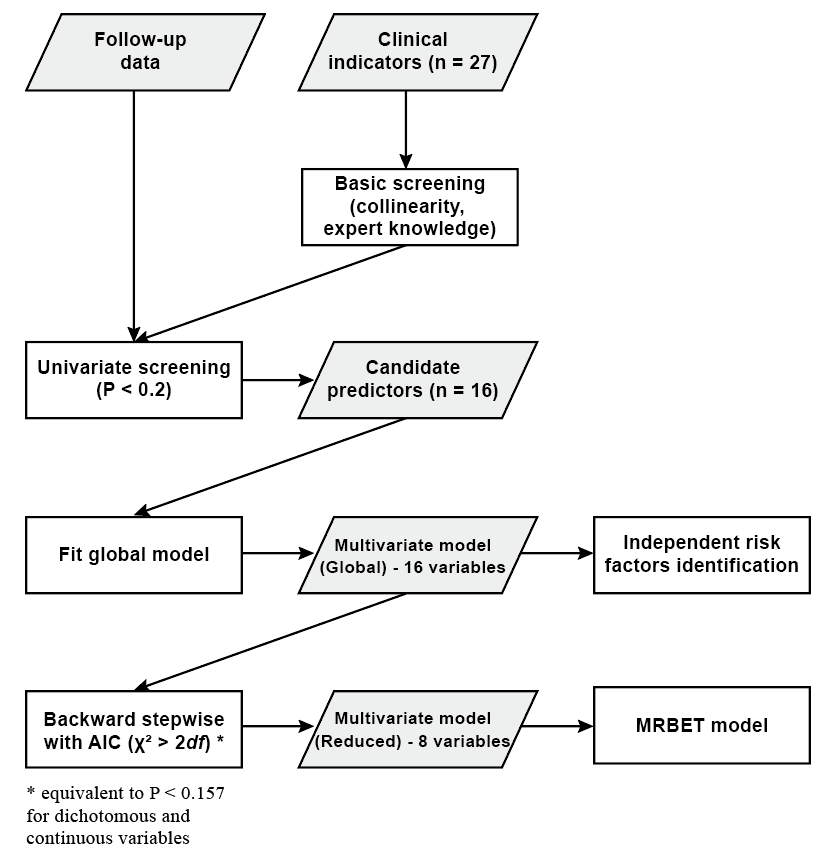


**Supplementary Fig. 1** The modeling process of this study.

Supplement: Supplementary file 1 — Supplementary file1 (DOCX 77 KB) [file 12072_2022_10464_MOESM1_ESM.docx]
